# Different influences of facial attractiveness on judgments of moral beauty and moral goodness

**DOI:** 10.1038/s41598-019-48649-5

**Published:** 2019-08-21

**Authors:** Xuan Cui, Qiuping Cheng, Wuji Lin, Jiabao Lin, Lei Mo

**Affiliations:** Center for Studies of Psychological Application, Guangdong Key Laboratory of Mental Health and Cognitive Science, School of Psychology, South China Normal University, Guangzhou, 510631 China

**Keywords:** Social behaviour, Morality

## Abstract

Are beauty and goodness the same? The relationship between beauty and goodness has long been a controversial issue in the fields of philosophy, aesthetics, ethics and psychology. Although many empirical studies have explored moral judgment and aesthetic judgment separately, only a few studies have compared the two. Whether these two judgments are two different processes or the same process with two different labels remains unclear. To answer this question, the present study directly compared the influence of facial attractiveness on judgments of moral goodness and moral beauty and revealed distinct contributions of imaging perceptions to these two judgments. The results showed that in the moral beauty judgment task, participants gave higher scores to characters with attractive faces compared with characters with unattractive faces, and larger P200 and LPP were elicited in the unattractive-face condition compared with the attractive-face condition; while in the moral goodness judgment task, there was no significant difference between the two conditions of either behaviour or ERP data. These findings offer important insights into the understanding and comparison of the processes of moral judgment and aesthetic judgment.

## Introduction

Goodness and beauty are two ultimate ends that humankind constantly pursues. These two concepts have been connected to each other since the dawn of civilization, and the debate continues regarding whether the two are the same^1–3^. Many great philosophers have shed light on this question^1,4^. For example, Aristotle argued that the goal of all virtue is beauty, while Kant considered beauty a symbol of moral goodness^1,4^.

The issue of the relationship between goodness and beauty has not been limited to philosophical discussions in ancient times; people can have their own experiences of this in daily life. Imagine the following scenario: An elderly man falls over the curb of a street, and a young girl passing by immediately runs to him and attempts to help. If you witnessed this, you would probably be impressed by the kindness of the girl’s deed, and it may also occur to you that this girl has a beautiful heart. During this time, you experience two different processes: You make a moral judgment regarding the goodness of the girl’s behaviour; and you make an aesthetic judgment regarding the moral beauty of the girl’s heart. These two judgments are based on the same scene, and both produce positive feelings. This phenomenon raises the following question: Are moral goodness judgments and moral beauty judgments the same process labelled with different names, or are they two different processes?

Researchers have conducted extensive empirical studies and accumulated valuable knowledge concerning the separate processes of moral judgment and aesthetics^5–10^. Moral judgment, by definition, is a process by which a person makes judgments of particular behaviours or options as being ethical/unethical or correct/incorrect^11^. This process also involves a tendency to see certain actions and individuals as right, good, and deserving of reward and others as wrong, bad, and deserving of punishment^12^. Traditional theories of moral psychology have adopted a rational view and suggested that moral judgment is mainly a reasoning process according to social rules^5,6^. People may make different moral judgments in response to the same question due to the development of high-level cognitive ability^7,8^. However, researchers have recently begun to emphasize the importance of affective processes for moral judgment. Haidt proposed the social intuitionist model and argued that moral judgment is not caused by reasoning but by rapid, affectively based intuitions and that moral behaviour covaries with emotion more than with reasoning^4,10,13,14^. Both behavioural and brain imaging evidence have confirmed the important role of emotion in moral judgment^15,16^. Greene and his collaborators proposed the dual-processing model, a synthetic theory of the above two viewpoints, which argued that social-emotional processing and cognitive processes are two essential elements of moral judgment^9,10^. Multiple neuroscientific findings have shown that the brain areas activated during moral judgments involve both areas related to emotion and areas related to cognitive processes, and these two elements both have considerable effects on moral judgment^17–19^. Such findings provide support for the above theory. Currently, moral judgment is mainly considered to be the result of both reasoning processing and affective processing.

Similar to the continuous scientific exploration of morality, beauty has been studied extensively^4,20,21^. According to aesthetic theory, the processes of aesthetic judgment involve a sensory-perceptual process, a cognitive process and an affective process^4,20,21^. In accordance with the above theory, the results of neuroaesthetic studies indicate that the brain areas involved in aesthetic judgment include the ventral visual systems [V1, V2, V4 and inferior temporal gyrus (ITG)], which are associated with visual processing, the superior frontal gyrus (SFG), which is associated with cognitive processing, and the orbitofrontal cortex (OFC), which is associated with affective processing^22^. With regard to these different processes, numerous findings emphasize the dramatic effect of objects’ audio-visual features on aesthetic assessment and suggest that the sensory and perceptual processing of an object’s image is not only the primary step in the process but is also crucial for making aesthetic decisions^23,24^. According to aesthetic theory, beauty can be further divided into three categories: natural beauty, artistic beauty and moral beauty. The first two, natural beauty and artistic beauty, are external beauty, the judgment of which is directly based on the processing of external auditory and/or visual features^4,20,21^, while the last one, moral beauty, refers to inner beauty^4,20,21^. Although external beauty has been the traditional subject of aesthetic studies, it is noteworthy that more recently, moral beauty has become the focus of a series of studies^13,25^. Moral beauty, according to its definition, is mainly the beauty of humanity, virtue and talent, which is evaluated based on the understanding of social regulations and involves social affect and advanced cognition^4,25,26^. In contrast to external beauty, moral beauty involves not only sensory and symbolic elements but also complex social implications^4,25,26^. One representative example of a situation in which the beauty promulgated by the mainstream media was actually moral beauty rather than merely external beauty was when the national television station of the People’s Republic of China, China Central Television, awarded a doctor who provided free treatment for orphans the title of “one of the most beautiful doctors in China”^27,28^. This award indicates the admiration of a beautiful inner character rather than an attractive appearance. Wang *et al*. performed fMRI experiments to explore moral beauty judgments^29^. Participants were asked to make aesthetic judgments and gender judgments of either face pictures or scene pictures describing good or neutral behaviour. The results showed that during moral beauty judgments, compared with the control condition, stronger brain activation was observed in the OFC (which is associated with affective processing), the ITG (which is associated with visual processing), and the SFG (which is associated with cognitive processing)^22^. These findings suggest that the process of moral beauty judgment, similar to external beauty judgment, also involves sensory-perceptual, cognitive and affective processes^22^.

A number of studies have further investigated the relationship between moral character and physical attractiveness. On the one hand, the results show that physical appearance has an effect on the evaluation of moral character, known as the beauty-is-good stereotype^30,31^. For instance, when participants were asked to view photographs of individuals first and then evaluate these individuals’ traits, attractive individuals were assumed to be more kind, honest, and moral compared with unattractive persons^30^. On the other hand, information on internal characteristics also has an impact on the judgment of external appearance, known as the good-is-beautiful stereotype^32,33^. For example, when participants were asked to evaluate the physical characteristics of a person shown in a head-and-shoulder photograph before reading a description of either high or low personality traits of the person, the honest target was considered to have a face that looked more attractive than the dishonest target^33^. Therefore, moral judgment and aesthetic judgment mutually affect each other. With the development of neuroimaging technology, researchers have adopted brain imaging methods to explore the neural features underlying the connection between moral and aesthetic judgments. Tsukiura *et al*. focused on the medial orbitofrontal cortex and insular cortex and tested whether similar activation patterns were found in these regions in response to two tasks: the attractiveness rating of facial photos and the goodness rating of short sentences that depicted actions^34^. They found that compared with the control conditions, the medial orbitofrontal cortex showed increased activity and the insular cortex showed decreased activity in both tasks. The researchers inferred that these two regions may underlie the mutual effect between aesthetic and moral judgments. Avram *et al*. employed one-line poems and short moral statements as stimuli to compare judgments of poetic beauty and judgments of a moral statement’s correctness. They discovered that the orbitomedial prefrontal cortex showed activation in both judgments, while areas such as the posterior cingulate cortex, the precuneus, and the temporoparietal junction were activated specifically in judgments of a moral statement’s correctness^22^.

In summary, abundant scientific investigations have been conducted separately on the processes of moral judgment and aesthetic judgment, and evidence indicates that there may be both similarities and distinctions between the neural bases of moral goodness and beauty^22,34^. However, no previous study has directly compared the process of moral judgment and the process of aesthetic judgment, and it remains unclear whether these two judgments are the same process with two different labels or two different processes. Moral beauty, compared with external beauty, is a type of beauty that is more abstract and more closely related to social affection and social cognition; thus, it is more easily confused with moral goodness^4,13,25^. Hence, an exploration of the similarities and differences between moral beauty and moral goodness will have valuable implications for answering the controversial question of the relationship between beauty and goodness. Based on reviews of previous studies of moral and aesthetic judgments, it can be concluded that moral goodness judgments mainly involve cognitive and affective processes^4,9,10^, while moral beauty judgments mainly involve sensory-perceptual, high-level cognitive and affective processes^22,29,35^. Both judgments include cognitive and affective processes, although the sensory-perceptual process of constructing images of evaluation targets for moral judgments has not been emphasized as much as it has for aesthetic judgment. Notably, a number of studies indicate that the perceived visual features of the input stimuli are decisive for generating aesthetic feelings and that sensory-perceptual processing ranks as a cardinal part of the aesthetic judgment. Thus, the judgment of moral beauty is based on the understanding of social rules, which is equally required in the moral goodness judgment; at the same time, the judgment of moral beauty also depends on the perception of the input stimuli and emphasizes the sensory-perceptual process, which is involved but not decisive in moral goodness judgments^29^.

The present experiment aimed to compare the process of evaluating moral beauty and the process of evaluating moral goodness using event-related potentials (ERPs) to explore similarities and differences between these two judgments. In accordance with the above analyses, we directly test whether different facial attractiveness affects the processes of moral beauty judgments and moral goodness judgments distinctly or similarly through a manipulation of attractive-face-version stimuli and unattractive-face-version stimuli. This exploration will provide new knowledge to resolve the long-standing dispute regarding the relationship between goodness and beauty. The ERP analysis focused on two ERP components, P2 and late positive potential (LPP). The P2 component is a positive component that peaks approximately 200 ms after the presentation of a stimulus^36^. It typically reflects the perceptual processing of stimuli^37,38^. Previous studies have shown that the peak amplitude of P2 is modulated by attention. It is purported that P2 represents the beginning of a central process responsible for stimulus identification and the initiation of decision making^39–41^. LPP is a positive component that occurs approximately 600 ms after stimuli onset and is maximal in the central and parietal regions. It is proposed to reflect advanced cognitive processes such as the conscious assessment of targets and deliberate reasoning^42,43^. For example, a larger LPP amplitude was found in the moral decision-making process when stronger conflicts existed^42,44^. It is hypothesized that for moral beauty judgments, in which the sensation and perception of an image play a decisive role in decisions, facial attractiveness will cause the separation of the P2 and subsequent LPP amplitudes, and the rating scores for attractive-face version stimuli with will be higher than the scores for unattractive-face version stimuli; while for moral goodness judgments, in which the attractiveness of images is not crucial for decisions, there will be no significant difference in P2 amplitudes, LPP amplitudes or rating scores between the two conditions.

## Results

### Behavioural results

**Figure 1:**
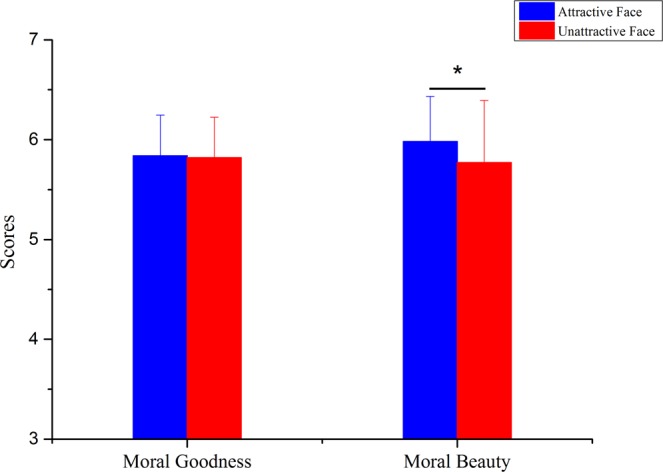
Rating scores of attractive-face-version stimuli and unattractive-face-version stimuli in the tasks of moral goodness judgment and moral beauty judgment. The results of rating scores demonstrated that the attractive-face-version stimuli received higher scores compared to the unattractive-face-version stimuli only in the moral beauty judgment. Error bars indicate the standard deviation of rating scores. *Indicates p < 0.05.

For the behavioural data, analyses of the rating scores and response times (RTs) were performed. With the rating score as the dependent variable, a two-way repeated-measures ANOVA was conducted with the judgment type (moral goodness judgment vs. moral beauty judgment) and facial attractiveness (attractive face vs. unattractive face) as within-subjects factors. The results, as demonstrated in Fig. 1, showed that the main effect of facial attractiveness was significant, F (1,21) = 5.012, P = 0.036, η2 = 0.193. The main effect of judgment type was not significant, F (1,21) = 0.263, P = 0.614, η2 = 0.012. There was an interaction between judgment type and facial attractiveness, F (1,21) = 4.750, P = 0.041, η2 = 0.184. The post-hoc analysis of the interaction revealed that in the moral goodness task, characters with attractive faces (m = 5.838, sd = 0.407) and characters with unattractive faces (m = 5.821, sd = 0.405) showed no difference in behavioural goodness, F (1,21) = 0.278, P = 0.604, η2 = 0.013. However, in the moral beauty task, characters with attractive faces (m = 5.982, sd = 0.450) were believed to show more moral beauty than characters with unattractive faces (m = 5.770, sd = 0.622), F (1,21) = 5.500, P = 0.029, η2 = 0.208.

**Figure 2:**
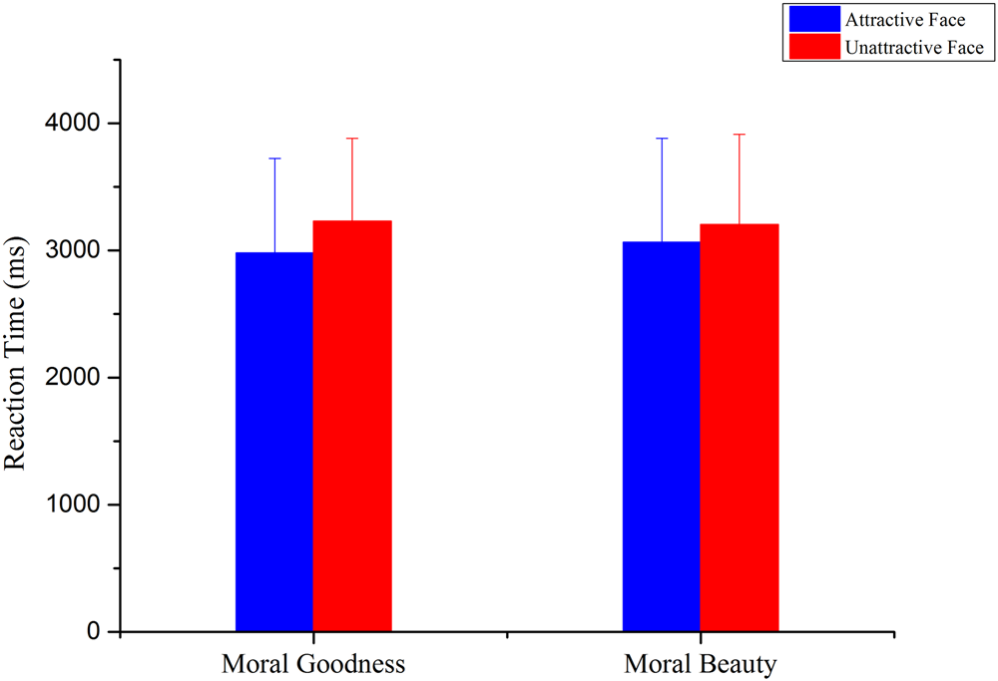
RTs of attractive-face-version stimuli and unattractive-face-version stimuli in tasks of moral goodness judgment and moral beauty judgment. The results of RTs showed no interaction between the judgment type and facial attractiveness. Error bars indicate standard deviation of RTs.

**Figure 3:**
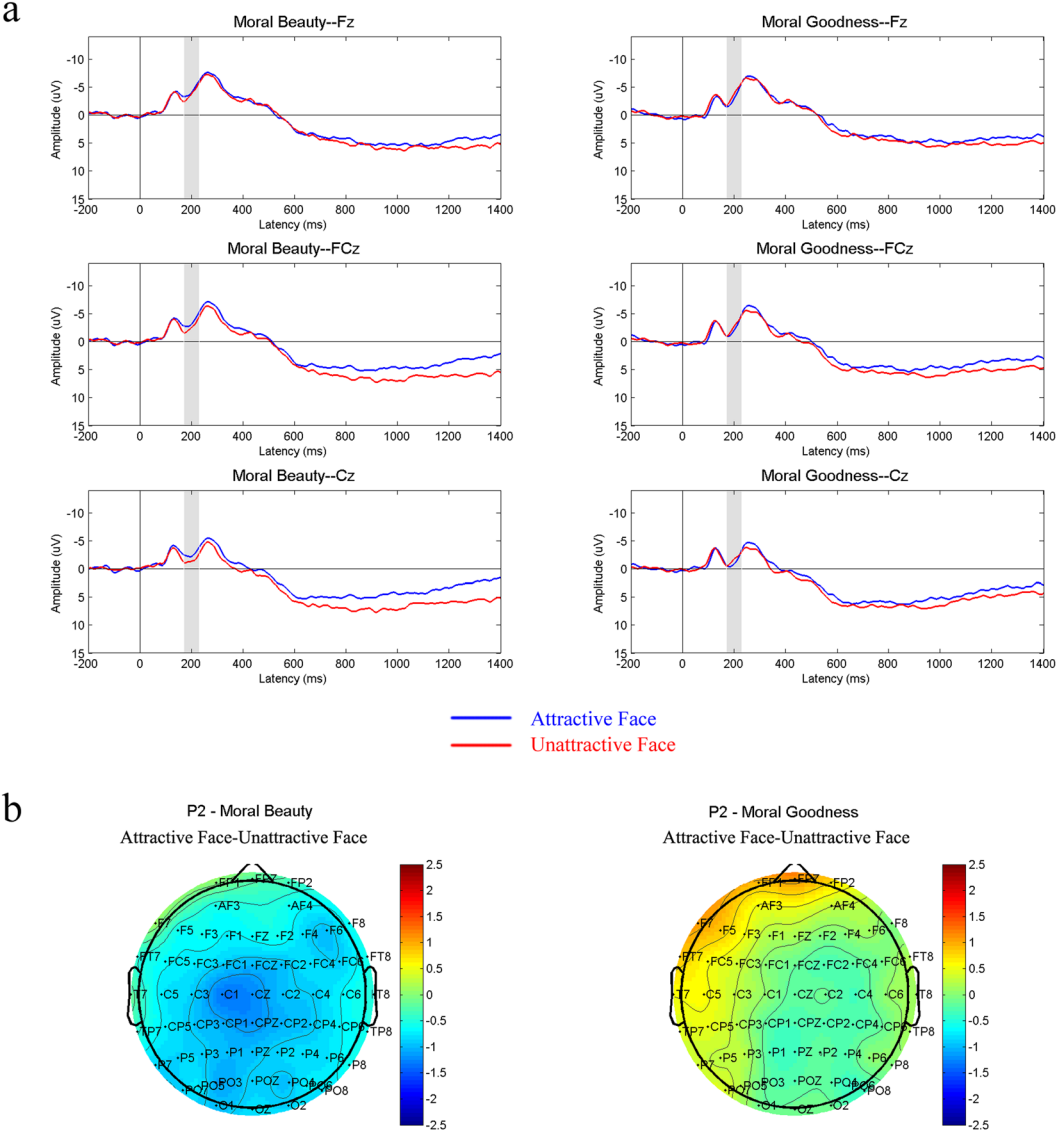
(**a**) The grand-average ERPs elicited by attractive-face-version stimuli and unattractive-face-version stimuli in tasks of moral beauty judgment and moral goodness judgment at midline recording sites, Fz, FCz and Cz. Shaded areas indicate the time range of the P2 component (170 ms-230 ms). (**b**) Topographic map illustrating the scalp distribution of the ERP amplitude difference between the two versions of stimuli (attractive face minus attractive face) in the P2 time window.

**Figure 4:**
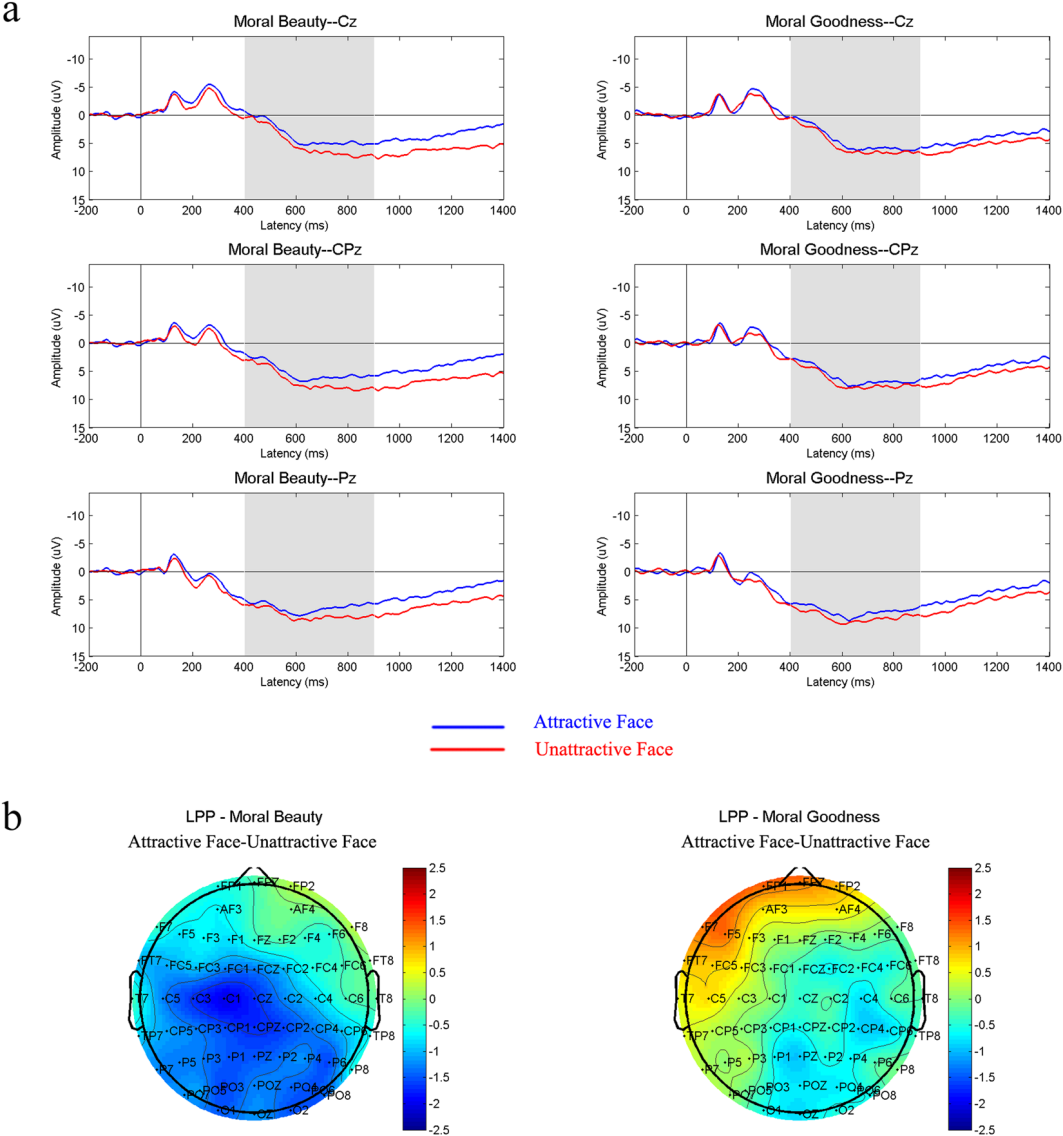
(**a**) Grand average ERPs elicited by attractive-face-version stimuli and unattractive-face-version stimuli in tasks of moral beauty judgment and moral goodness judgment at midline recording sites, Cz, CPz and Pz. Shaded areas indicate the time range of the LPP component (400 ms–900 ms). (**b**) Topographic map illustrating the scalp distribution of the ERP amplitude difference between two versions of stimuli (attractive face minus attractive face) in the LPP time window.

With the RTs as the dependent variable, a two-way repeated-measures ANOVA was conducted with judgment type (moral goodness judgment vs. moral beauty judgment) and facial attractiveness (attractive face vs. unattractive face) as within-subjects factors. The results, as demonstrated in Figs 2, 3, 4, showed that there was no interaction between the judgment type and facial attractiveness, F (1,21) = 0.478, P = 0.497, η2 = 0.022. The main effect of judgment type was not significant, F (1,21) = 0.027, P = 0.870, η2 = 0.001. The main effect of facial attractiveness was significant, F (1,21) = 6.346, P = 0.020, η2 = 0.232, indicating that drawings of characters with attractive faces were rated more quickly (m = 3019.87 ms, sd = 774.519) than drawings of characters with unattractive faces (m = 3214.66 ms, sd = 674.173).

### ERP Results

#### P2

In accordance with previous studies^37,45^, the mean amplitudes of nine frontal and central electrode sites (F3, Fz, F4, FC3, FCz, FC4, C3, Cz, and C4) were measured in a 170–230 ms time window as P2 amplitudes. The P2 results were showed in Figure 3. With P2 amplitudes as the dependent variable, a two-way repeated-measures ANOVA was conducted with judgment type (moral goodness judgment vs. moral beauty judgment) and facial attractiveness (attractive face vs. unattractive face) as within-subjects factors. The results showed that the main effect of judgment type was not significant, F (1,21) = 0.782, P = 0.387, η2 = 0.036. The main effect of facial attractiveness was not significant, F (1,21) = 2.044, P = 0.168, η2 = 0.089. There was an interaction between judgment type and facial attractiveness, F (1,21) = 9.794, P = 0.005, η2 = 0.318. The post-hoc analysis of the interaction further revealed that in the moral goodness task, characters with attractive faces (m = −2.135, sd = 3.008) and characters with unattractive faces (m = −2.443, sd = 3.637) showed no difference in the moral goodness judgment task, F (1, 21) = 0.533, P = 0.473, η2 = 0.025. In the moral beauty task, the P2 amplitudes elicited by stimuli with facially unattractive characters (m = −1.922, sd = 3.382) were significantly larger than those elicited by stimuli with facially attractive characters (m = −3.102, sd = 2.958), F (1,21) = 11.466, P = 0.003, η2 = 0.353.

#### LPP

In accordance with previous studies^46,47^, the mean amplitudes of nine central and parietal electrode sites (C3, Cz, C4, CP3, CPz, CP4, P3, Pz and P4) were measured in a 400–900 ms time window as LPP amplitudes. The LPP results were showed in Figure 4. With LPP amplitudes as the dependent variable, a two-way repeated-measures ANOVA was conducted with judgment type (moral goodness judgment vs. moral beauty judgment) and facial attractiveness (attractive face vs. unattractive face) as within-subjects factors. The results demonstrated that the main effect of judgment type was not significant, F (1,21) = 0.747, P = 0.397, η2 = 0.034. The main effect of facial attractiveness was significant, F (1,21) = 11.801, P = 0.002, η2 = 0.360. There was an interaction between judgment type and facial attractiveness, F (1,21) = 6.888, P = 0.016, η2 = 0.147. The post-hoc analysis of the interaction revealed that in the moral goodness task, characters with attractive faces (m = 4.268, sd = 3.143) and characters with unattractive faces (m = 4.811, sd = 3.540) showed no difference in behavioural goodness, F (1,21) = 1.170, P = 0.292, η2 = 0.053. In the moral beauty task, LPP amplitudes elicited by stimuli with facially unattractive characters (m = 5.881, sd = 3.265) were significantly larger than those elicited by stimuli with facially attractive characters (m = 3.881, sd = 3.359), F (1,21) = 22.730, P < 0.001, η2 = 0.520.

## Discussion

The present study investigated whether judgments of moral goodness and moral beauty are two distinct judgment processes or are actually the same process with two different labels. To answer this question, participants were presented with a series of moral behaviour scene drawings in which the character had either an attractive face or an unattractive face. The participants completed two tasks, the moral goodness judgment task and the moral beauty judgment task, separately while ERP signals were recorded throughout the experiment to compare whether the character’s facial attractiveness had the same impact on the moral goodness judgments and the moral beauty judgments.

The results of the rating scores showed that for the moral beauty judgment, although all pictures presented in the experiment depicted positive deeds, scene drawings with attractive-face characters received higher scores compared with scene drawings with unattractive-face characters. In contrast, in the moral goodness judgment, scene drawings with attractive-face characters and scene drawings with unattractive-face characters showed no significant difference. Overall, the behavioural results indicate that the facial attractiveness of the character had a notable influence on the moral beauty assessment but did not have a significant impact on the moral goodness assessment.

The results of the two ERP components demonstrated a similar pattern as the behavioural findings, as hypothesized. For the evaluation of moral beauty, the variable of the facial attractiveness of the character caused the separation of the amplitudes of P2 and LPP, while there was no significant difference between the two versions of the material for the moral goodness rating. Both behavioural and ERP data were collected and analysed to maximize the reliability of the present study. The convergence of these two types of evidence validated the findings of this study.

Previous studies have shown that P2 is not only related to the detection of visual features but also reflects higher-order perceptual processing^36,48^. Its amplitudes are modified by attention and other cognitive variables^39–41,48^. It has been proposed that P2 represents the beginning of a central process responsible for stimulus identification and the initiation of decision making^49^. The P2 results demonstrated that in the task of moral beauty judgment, the variable of characters’ facial attractiveness modulated the P2 amplitude but showed no significant effect in the same time window in the moral goodness judgment task. These findings indicate that the variable of facial attractiveness affected the processing of moral beauty decisions but showed no evident influence on the processing of moral goodness decisions. Considering the matched stimuli used for these two tasks, this distinction is believed to be due to the different requirements of the moral goodness judgment and the moral beauty judgment. In the former task, when participants integrate the visual information of the scene drawings to make a moral goodness judgment as instructed, whether a character has an attractive or unattractive face may not be the decisive information for evaluating the goodness of the character’s behaviour. Thus, this variable had a limited influence and caused no significant difference in the P2 amplitudes between the attractive-face and unattractive-face conditions. In the latter task, however, when the participants viewed the scene drawings to assess the beauty of the character’s heart as instructed, although the character’s behaviour was one important element to consider, the impression of the character’s appearance was also influential for the final moral beauty rating. Thus, different levels of facial attractiveness caused the separation of the P2 amplitudes. In contrast, for the moral goodness judgment, the attractive-face stimuli and the unattractive-face stimuli showed no evident difference in P2 amplitudes. Previous studies have indicated that facial attractiveness may have an impact on the evaluation of personality traits^30,31,50^. For example, individuals with high attractiveness (compared with those with low facial attractiveness) are less likely to be considered guilty and more likely to be judged as having positive characteristics, such as kindness and honesty^30,50^. However, in the present study, in which the instructions for the moral goodness judgment specifically asked participants to evaluate the goodness of the characters’ behaviour, the influence of facial attractiveness on the goodness judgment was limited and thus caused no significant difference between the two versions of the stimuli.

The LPP results showed a similar pattern as the P2 results. The characters’ appearance influenced the participants’ neural responses to the moral beauty evaluation of the scene drawings but showed no such effect in the moral goodness rating of matched scene drawings. Previous ERP studies have demonstrated that LPP reflects slow but elaborate moral reasoning processes and aesthetic evaluation processes^42,44^. LPP is thus considered a neural index of the later cognitive process during reasoning and decision-making that is involved in both moral judgment and aesthetic judgment processes^42,44^. In accordance with the above analysis, it is believed that LPP mainly reflects the cognitive reasoning process of moral and aesthetic judgments. In the present experiment, this refers specifically to reasoning and decision making based on the synthetic scene information formed during the perception stage. The present results show that during the task of making moral beauty decisions, the separation of the P2 component indicates that characters’ different external facial attractiveness had an impact on the early stage of the evaluation process. The separation of LPP, similar to the pattern for P2, revealed that the image-constricting process further influences the assessment of moral beauty at later time points. The same variable showed no evident influence on neural responses during moral goodness judgments at either early or late latencies. It is inferred that in the process of moral beauty judgment, the character’s appearance information is especially noticed at the early stage, which produces a significant difference in the P2 amplitude under the two different conditions. When participants further integrated the information on a character’s facial features and information on the character’s behaviour to make a comprehensive moral beauty judgment, different perceptions of the character’s facial attractiveness caused further differences when combined with the same positive behaviour in the final stage of providing a moral beauty score, as demonstrated by the significant difference between the two versions of stimuli in the behavioural results and LPP amplitudes. The relatively larger LPP amplitudes of the unattractive-face condition in the moral beauty judgment were believed to occur because of the inconsistency of information. When evaluating the moral beauty of characters with attractive faces, the participants could smoothly combine an attractive face and good behaviour to obtain positive results through a consistent cognitive reasoning process. In contrast, when evaluating moral beauty with the unattractive-face material, a lack of conformity occurred. If participants were asked to solely assess the beauty of a series of unattractive faces or the goodness of a set of noble deeds, the answer would naturally be negative or positive. However, when participants were required to evaluate the moral beauty of characters with unattractive faces who performed noble deeds, the diversity of the decision tendencies could cause cognitive conflict, which further elicited a larger LPP amplitude compared with the consistent condition in which participants viewed attractive-face characters performing positive behaviours. Previous studies have shown that an inconsistent condition leads to greater LPP amplitudes than a consistent condition^51–53^. The findings of this study are in accordance with these findings.

In contrast to the moral beauty judgment, for the moral goodness judgment, with external facial attractiveness information did not receive sufficient attention in the early stage, as reflected in the fact that the attractive and unattractive faces did not lead to the separation of P2 components, the further influence of facial attractiveness in the later stage of cognitive processing was limited. Thus, at later time points, the LPP induced by the two different conditions, the attractive-face condition and the unattractive-face condition, demonstrated no significant difference. These distinct results of the LPP amplitudes between moral goodness and moral beauty judgments reveal that perceived facial attractiveness information in the sensation and perception stages influences later stages of moral beauty assessment, but similar effects are not observed in the moral goodness evaluation. This finding indicates the unmatched influence of sensory and perceptual processing on judgments of moral goodness and moral beauty.

Combining results of the behavioural and ERP data, it is evident that facial attractiveness has different effects on the processes of moral goodness and moral beauty judgments from the early stage to the late stage. These results indicate the dissimilar influence of the perceived image on the evaluation of moral goodness and moral beauty, suggesting a distinction between the processes of judgments of moral goodness and moral beauty. One crucial factor that distinguishes them lies in the divergent impact of the sensory and perceptual construction of the image on the processes of the two judgments. The salient influence of a character’s facial attractiveness on judgments of moral beauty is consistent with the theory of aesthetic process, which argues that aesthetic judgments engage a two-stage process: the earlier stage is impression formation, and the later stage is evaluative aesthetic categorization^54^. Many studies have accordingly indicated that objective audio-visual feature evaluation occurs first, followed by aesthetic decision making, which is affected by both the result of the former descriptive judgment and a complex set of factors, such as emotional factors and appeals to social status.

The present study aimed to directly compare the influence of facial attractiveness on judgments of moral goodness and moral beauty. With this goal, scene drawings were chosen as suitable experimental materials for they have a significant advantage of simultaneously providing information on both characters’ external facial attractiveness and behaviour. Many studies have indicated that both aesthetic judgment and moral judgment can be intuitive processes, and people make these decisions rapidly^13,55–57^. In contrast to previous studies that presented words, words and facial photographs or facial photographs and scene drawings separately on different slides^22,29,34^, in the present experiment, information on both external facial attractiveness and behaviour was contained in one scene drawing and presented to participants at the same time, which enabled a more natural judgment process. For future studies, more material options, such as photographs of real moral scenes and characters and video clips that record real moral events, could be used to explore whether different types of stimuli affect the results and to test the robustness of the present findings. In addition, as previous studies have demonstrated that multiple factors, such as symmetry, averageness, sexual dimorphism and skin colour/texture, systematically affect the perception of facial attractiveness^58–60^, future studies could explore whether these factors function differently with regard to the effect of facial attractiveness on judgments of moral beauty and moral goodness.

Another reason why the present study concentrated on the investigation of positive judgments (moral goodness judgments and moral beauty judgments) is that compared to the large numbers of moral judgment studies that focus on participants’ negative evaluations of morally bad behaviours, such as violations of moral norms and maleficent behaviours^19,61,62^, fewer studies focus on people’s positive assessments of morally good behaviours and this process is much less clear^29,34,63^. Studies of judgments of moral goodness and moral beauty are needed to fill this theoretical gap. In addition, such studies have practical significance and can serve as the basis for designing a more scientific and efficient way to conduct moral education^26,63^. The investigation of the relationship between moral and aesthetic judgments is far from complete. Previous findings have shown that moral goodness judgments and moral badness judgments may have distinct neural mechanisms and functional connectivity^64–66^. For example, one study found that judgments of scenarios classified as morally good (compared with judgments of morally bad scenarios) involved greater activity in areas of the middle and inferior temporal gyri, inferior frontal gyrus, dorsolateral prefrontal cortex and precuneus. In contrast, judgments of morally bad scenarios(compared with judgments of morally good scenarios) involved greater activity in areas of the hippocampus and midcingulate cortex, superior frontal gyrus, supramarginal gyrus, left inferior and superior parietal lobules, and insula. Thus, it would be interesting and important to further explore whether the influence of facial attractiveness on judgments of moral badness and moral ugliness would show different patterns from judgments of moral goodness and moral beauty and obtain a more comprehensive understanding of moral and aesthetic judgments. Furthermore, with the development of neuroscience technologies such as transcranial magnetic stimulation (TMS) and transcranial direct current stimulation (tDCS) that temporarily change the brain function of certain areas^67,68^, a more thorough investigation could be conducted by specifically changing the function of certain brain areas (such as areas related to facial perception) and exploring the modulation caused by this manipulation on the processes of moral judgments and aesthetic judgments. This investigation could provide a more advanced way to understand and compare these processes.

In summary, to the best of our knowledge, the present research is the first to use validated scene drawings to present both facial attractiveness information and behavioural information at the same time and to directly compare the processes of moral beauty and moral goodness judgments. With the analysis of both behavioural and ERP data, the present work provides novel and compelling evidence on the difference between judgments of moral goodness and moral beauty and advances current knowledge on the detailed processes of moral and aesthetic judgments. It can be concluded that moral judgment and aesthetic judgment are different processes with two different labels. These findings offer important insights into the ongoing controversial debate about the relationship between beauty and goodness.

## Methods

### Participants

Twenty-two undergraduate students (mean age = 21 years, range, 18–24 years; 14 females) with no history of neurological or psychiatric problems were recruited as participants. Prior to initiating data collection, a power analysis was conducted in G*Power^69^ to determine the sample size that would provide sufficient power to detect a large effect size^70^. In accordance with previous studies, the power analysis revealed that for an effect size (η2) of 0.14 to be detected with an 80% chance of significance with the alpha level set to 0.05, the required sample would be 22 participants. Thus, the sample size of the present study was 22 participants. All participants were assessed before the experiment to ensure that they had normal or corrected-to-normal vision. The study was approved by the ethical review board of the Department of Psychology, South China Normal University and all volunteers gave written informed consent. All methods used in the current study were performed in accordance with the relevant guidelines and regulations of the ethical review board.

### Stimuli

**Figure 5:**
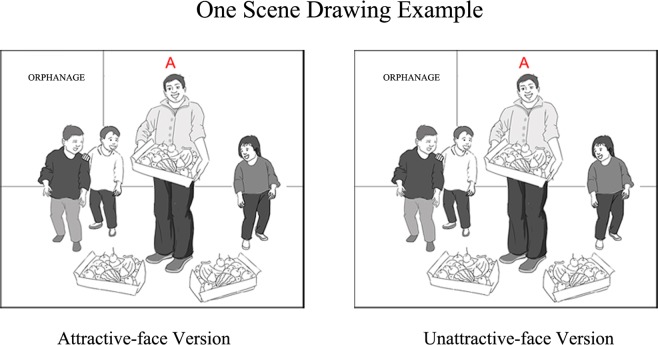
Examples of scene drawings. There are two versions of the character’s facial features for each scene: an attractive-face version (left) and an unattractive-face version (right).

The materials used in this experiment were scene drawings. Seventy-two scene drawings were created, validated and used as stimuli in the moral goodness judgment task and the moral beauty judgment task. In accordance with previous studies^29,71,72^, 80 short sentences that described good deeds, such as taking an injured old man to the hospital and visiting orphans in the orphanage, were collected. Then, black-and-white scene drawings were created based on the content of these sentences (see Fig. 5). A capital letter A was drawn beside the character to distinguish the character from other figures shown in the scene drawing. Finally, for each scene, there were two versions of the character’s facial features: an attractive-face version in which the faces of the characters were drawn to be attractive and an unattractive-face version in which the faces of the characters were drawn to be unattractive. The manipulation of facial attractiveness was not limited to the systematic change of a certain facial feature but rather to the random combination of several facial features.

Two pilot studies were conducted to validate the scene drawings. A total of forty-two participants were recruited. Twenty of them were asked to evaluate the goodness of the character’s behaviour described in the scene drawings on a 7-point scale (1 = extremely bad, 7 = extremely good), with the characters’ faces blurred to prevent the influence of facial features on the goodness evaluation. According to the results, scene drawings with average scores less than 4 were excluded to ensure that all behaviours described in the scene drawings were positive. The other twenty-two participants were asked to assess the attractiveness of characters’ faces in the scene drawings on a 7-point scale (1 = extremely ugly, 7 = extremely beautiful), with other parts of the pictures blurred to avoid the impact of the scene and the characters’ behaviour on the facial attractiveness assessment. Based on the results, drawings with average scores less than or equal to 4 were excluded for the attractive-face version scene drawings, and average scores greater than or equal to 4 were excluded for the unattractive-face version scene drawings. A total of 36 moral scenes were finally selected. Each scene had one attractive-face version and one unattractive-face version, composing 72 validated scene drawings that were used as final stimuli.

The 36 moral scenes were then randomly assigned to two sets. Participants made the moral goodness judgment on one stimuli set and made the moral beauty judgment on the other stimuli set to avoid the repeated use of the same stimuli in two different tasks. Each set contained eighteen moral scenes. As each scene had an attractive-face version and an unattractive-face version, each set consisted of 36 moral scene drawings in total. One independent-sample t test was conducted on the scores of the two sets, which were acquired from the first pilot study in which the participants specifically evaluated the goodness of the character’s behaviour described in the scene drawings. The results showed that there was no significant difference between the two sets (t = 0.397, p = 0.694). This result indicated that with regard to the degree of goodness depicted in the drawings, the two sets were equivalent. Similarly, two more independent-sample t tests were conducted separately on the facial attractiveness scores of the attractive-face-version stimuli of the two sets and the unattractive-face-version stimuli of the two sets. The facial attractiveness scores were the scores acquired from the second pilot study in which participants specifically assessed the facial attractiveness of the character with all content other than the characters’ face blurred. The results showed that there was no significant difference between the attractive-face version of the two stimuli sets (t = 0.677, p = 0.503) and the unattractive-face version of the two stimuli sets (t = 1.031, p = 0.310). All of the results showed no significant difference between the two sets, which ensured that the two sets were equivalent.

Thus, for each judgment task, eighteen moral scenes were used that contained eighteen attractive-face scene drawings and eighteen unattractive-face scene drawings. Every scene drawing was shown three times. Therefore, there were 54 trials for each experimental condition: The attractive-face-version stimuli in the moral goodness judgment, the unattractive-face-version stimuli in the moral goodness judgment, the attractive-face-version stimuli in the moral beauty judgment and the unattractive-face-version stimuli in the moral beauty judgment.

### Procedures

**Figure 6:**
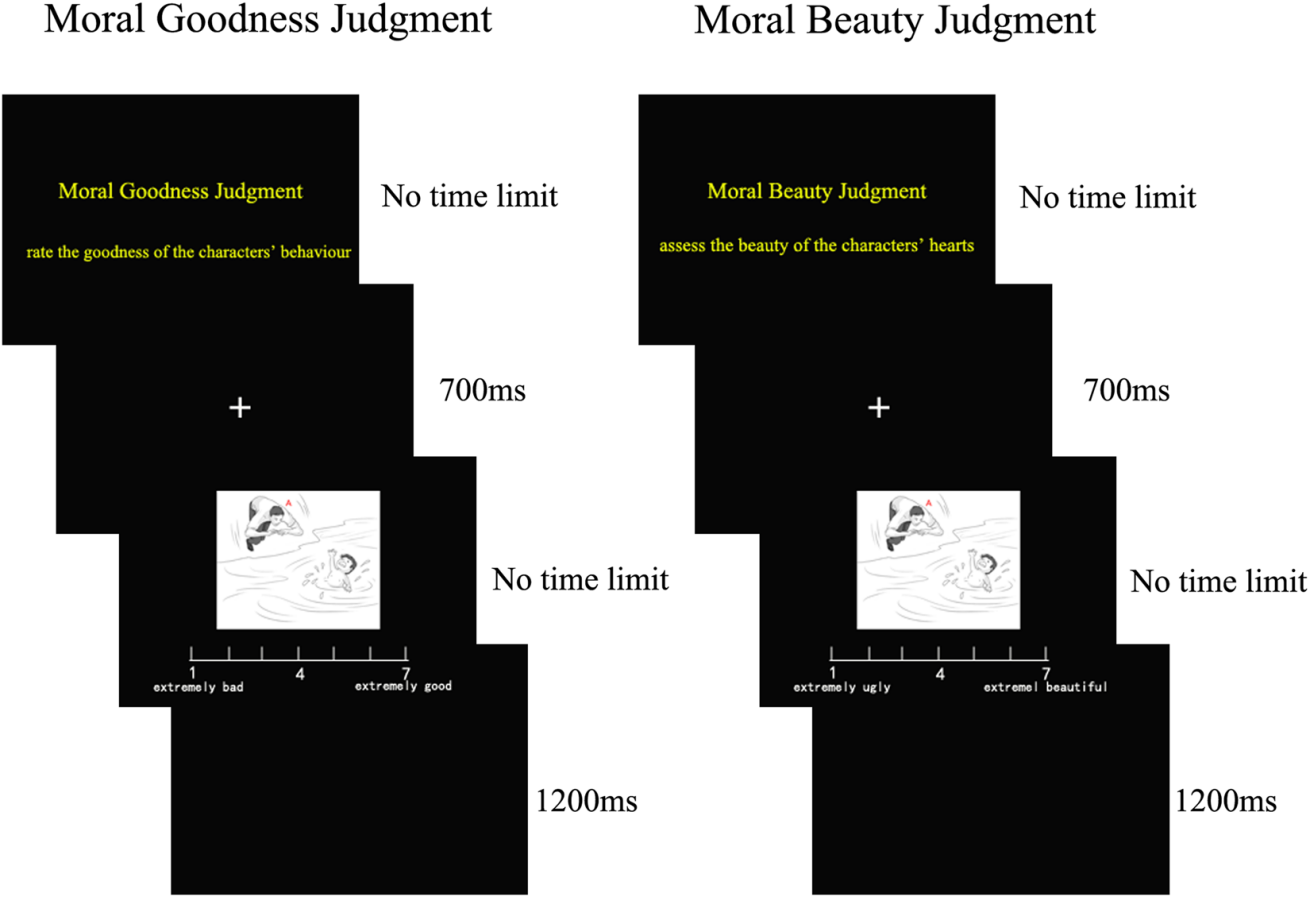
Experimental procedure. Each participant performed two tasks: The moral goodness judgment task and the moral beauty judgment task. For each trial, a fixation cross was presented at the centre of the screen for 700 ms, and then a scene drawing and a 7-point scale were shown until the participant pressed the number key in response. This was followed by a 1200 ms blank screen before the next trial began.

During the experiment, participants were seated in a sound-attenuated, electrically shielded, dimly illuminated chamber. Stimuli were presented in the centre of a 15-inch computer screen at a viewing distance of 85 cm to ensure that both the horizontal and vertical visual angles were less than 3°. The participants were asked to perform two types of tasks. One task was the moral goodness judgment, in which the participants were instructed to look at the scene drawings and evaluate the goodness of the character on a 7-point scale (1 = extremely bad, 7 = extremely good). The other task was the moral beauty judgment, in which the participants were instructed to look at the scene drawings and assess the beauty of the character’s heart on a 7-point scale (1 = extremely ugly, 7 = extremely beautiful). Two equivalent sets that were used separately in the moral goodness and moral beauty judgments were balanced across participants. The experiments included 3 runs of moral goodness judgments and 3 runs of moral beauty judgments. The order of the 6 runs was random, and each run consisted of eighteen trials. A single trial began with a fixation cross presented at the centre of the black background for 700 ms. Next, a scene drawing and a 7-point scale were shown on the screen. This slide did not disappear until the participant pressed the number key in response. Then, there was a 1200 ms blank screen before the next trial began (see Fig. 6).

### ERP recording and analysis

Continuous recordings of the electroencephalogram (EEG) were obtained throughout the experiment using an electrode cap that contained 64 electrodes positioned according to the International 10 ⁄ 20 System. For the purpose of artefact scoring, electrooculograms (EOGs) were also recorded. The horizontal electrooculogram (HEOG) was recorded using two electrodes placed lateral to the right and left eyes. The vertical electrooculogram (VEOG) was recorded with electrodes placed below the right eye.

During the off-line analyses, all data were visually scored for artefacts. Ocular artefacts were removed from the data using a regression-based correction algorithm implemented with the Neuroscan software. EEG signals were re-referenced to the average of the left and right mastoid recordings and low-pass filtered below 30 Hz. The EEG was segmented into 1600 ms epochs from 200 ms before to 1400 ms after the scene drawing stimulus onset. The EEG epochs were baseline corrected against the mean voltage during the 200 ms pre-stimulus period. Trials containing EEG sweeps with amplitudes exceeding ± 100 µV were excluded from the analysis.

## Data Availability

All data analyzed in this study are available from the corresponding author upon reasonable request.
