# Author Correction: Different influences of facial attractiveness on judgments of moral beauty and moral goodness

**DOI:** 10.1038/s41598-019-53743-9

**Published:** 2019-11-20

**Authors:** Xuan Cui, Qiuping Cheng, Wuji Lin, Jiabao Lin, Lei Mo

**Affiliations:** Center for Studies of Psychological Application, Guangdong Key Laboratory of Mental Health and Cognitive Science, School of Psychology, South China Normal University, Guangzhou, 510631 China

Correction to: *Scientific Reports* 10.1038/s41598-019-48649-5, published online 21 August 2019

In Figure 5, the character’s facial features for unattractive-face version are incorrect. The correct Figure 5 appears below as Figure 1.

**Figure 1:**
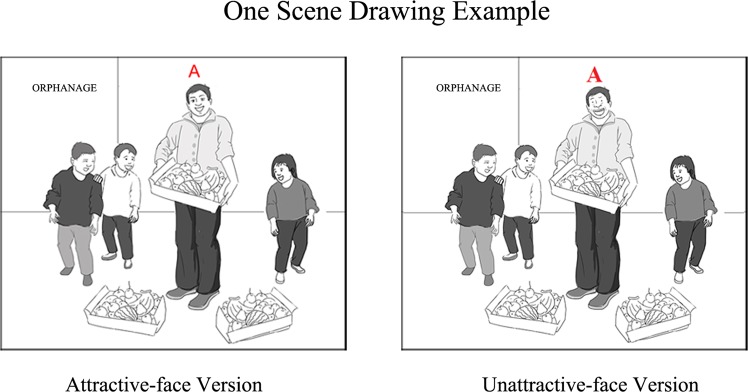
.

